# Are people with metabolically healthy obesity really healthy? A prospective cohort study of 381,363 UK Biobank participants

**DOI:** 10.1007/s00125-021-05484-6

**Published:** 2021-06-10

**Authors:** Ziyi Zhou, John Macpherson, Stuart R. Gray, Jason M. R. Gill, Paul Welsh, Carlos Celis-Morales, Naveed Sattar, Jill P. Pell, Frederick K. Ho

**Affiliations:** 1 Institute of Health and Wellbeing, University of Glasgow, Glasgow, UK; 2 Institute of Cardiovascular and Medical Sciences, University of Glasgow, Glasgow, UK; 3 Human Performance Laboratory, Education, Physical Activity and Health Research Unit, University Católica del Maule, Talca, Chile

**Keywords:** Cardiovascular disease, Metabolically healthy obesity, Obesity

## Abstract

**Aims/hypothesis:**

People with obesity and a normal metabolic profile are sometimes referred to as having ‘metabolically healthy obesity’ (MHO). However, whether this group of individuals are actually ‘healthy’ is uncertain. This study aims to examine the associations of MHO with a wide range of obesity-related outcomes.

**Methods:**

This is a population-based prospective cohort study of 381,363 UK Biobank participants with a median follow-up of 11.2 years. MHO was defined as having a BMI ≥ 30 kg/m^2^ and at least four of the six metabolically healthy criteria. Outcomes included incident diabetes and incident and fatal atherosclerotic CVD (ASCVD), heart failure (HF) and respiratory diseases.

**Results:**

Compared with people who were not obese at baseline, those with MHO had higher incident HF (HR 1.60; 95% CI 1.45, 1.75) and respiratory disease (HR 1.20; 95% CI 1.16, 1.25) rates, but not higher ASCVD. The associations of MHO were generally weaker for fatal outcomes and only significant for all-cause (HR 1.12; 95% CI 1.04, 1.21) and HF mortality rates (HR 1.44; 95% CI 1.09, 1.89). However, when compared with people who were metabolically healthy without obesity, participants with MHO had higher rates of incident diabetes (HR 4.32; 95% CI 3.83, 4.89), ASCVD (HR 1.18; 95% CI 1.10, 1.27), HF (HR 1.76; 95% CI 1.61, 1.92), respiratory diseases (HR 1.28; 95% CI 1.24, 1.33) and all-cause mortality (HR 1.22; 95% CI 1.14, 1.31). The results with a 5 year landmark analysis were similar.

**Conclusions/interpretation:**

Weight management should be recommended to all people with obesity, irrespective of their metabolic status, to lower risk of diabetes, ASCVD, HF and respiratory diseases. The term ‘MHO’ should be avoided as it is misleading and different strategies for risk stratification should be explored.

**Graphical abstract:**

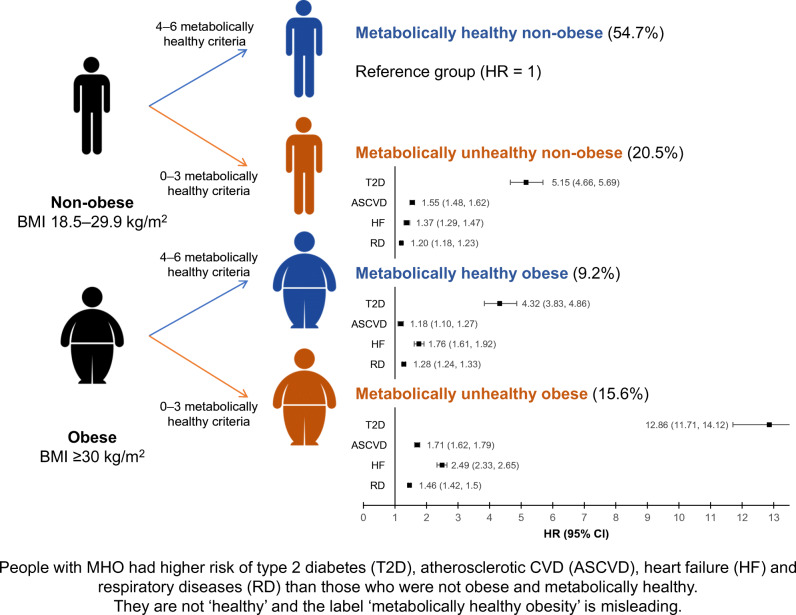

**Supplementary Information:**

The online version contains peer-reviewed but unedited supplementary material available at 10.1007/s00125-021-05484-6.

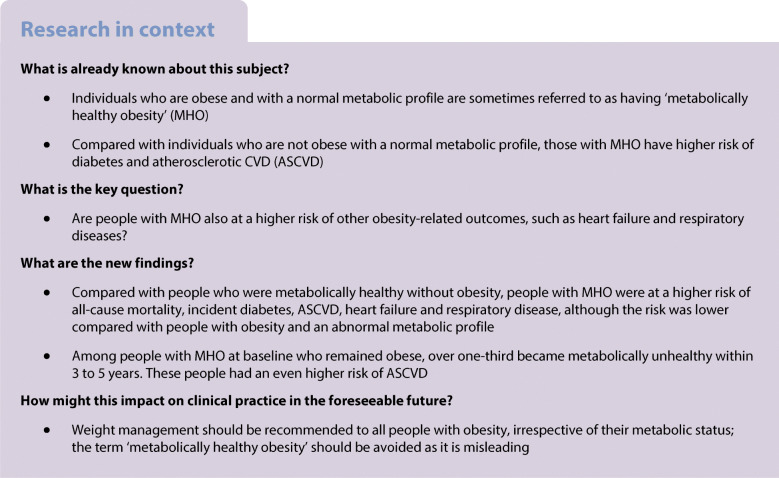



## Introduction

Over 300 million people are estimated to be obese worldwide [1]. Based on current trends, it is estimated there will be 1 billion obese people by 2030, accounting for 20% of the world’s adult population [1, 2]. Obesity is the main cause of the current global epidemics of type 2 diabetes, hypertension, CVD and many other diseases [3, 4].

Typically, obesity leads to metabolic dysfunctions such as elevated blood glucose, increased BP, dyslipidaemia (characterised by high triacylglycerols and lower HDL-cholesterol levels), systemic inflammation and insulin resistance [5]. However, some people with obesity have normal BP, favourable lipid and inflammatory profiles, and high insulin sensitivity [6, 7]. This phenotype is sometimes referred to as ‘metabolically healthy obesity’ (MHO) [6, 7]. The prevalence of MHO is estimated to be 3% to 22% in the general population [8, 9].

People with MHO have been shown to have an elevated risk of diabetes [10], but the evidence on atherosclerotic CVD (ASCVD) and all-cause mortality is mixed [11–13]. There are multiple reasons behind these mixed results. First, the definitions of MHO were inconsistent across studies [14]. Some studies defined MHO as participants who fulfil almost all of the criteria, resulting in a lower prevalence of MHO with similar risk to that in the general non-obese population [14]. It has been suggested that MHO may be a transitional state, rather than a distinct and stable phenotype [15, 16], and that people with MHO eventually develop metabolic dysfunction known as metabolically unhealthy obesity (MUO) [15]. At this stage, they have a higher risk of ASCVD and all-cause mortality, similar to those who originally had MUO. Importantly, research so far has focused on the association of MHO with ischaemic heart disease, stroke, diabetes and all-cause mortality, but has omitted other important obesity-related conditions such as respiratory diseases or heart failure (HF) [14]. In addition, even though diabetes is often thought of as a mediator between obesity and cardiovascular outcomes, there has been no study examining the mechanistic role of diabetes between MHO and CVD.

This study aimed to address the limitations of earlier research and determine the association of MHO, as well as its transition, with all-cause mortality, diabetes, ASCVD, HF and respiratory diseases. This study further explored to what extent diabetes mediates the association between MHO and cardiovascular outcomes.

## Methods

### Study design and participants

**Fig. 1:**
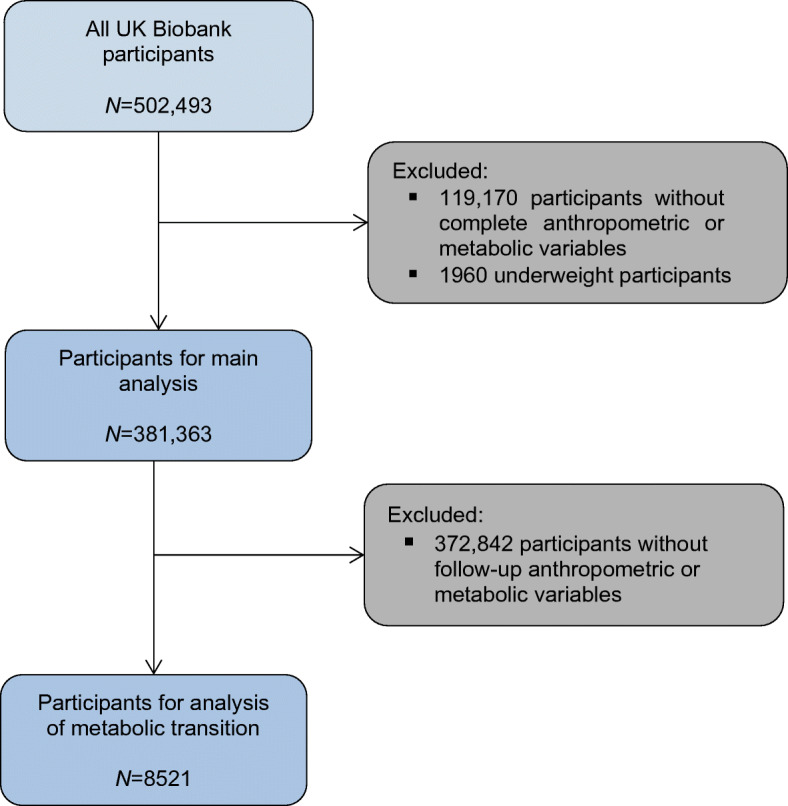
Participant flowchart

UK Biobank is a prospective cohort study. Between 2007 and 2010, UK Biobank recruited 502,493 participants from the general population [17]. Participants attended one of 22 assessment centres across England, Scotland and Wales where they completed a self-administered, touch-screen questionnaire and face-to-face interview, and trained staff took a series of measurements including height, weight and BP. This study included only the 381,363 participants who were not underweight and had complete data on height, weight, BP and blood-based biomarkers (Fig. 1). The analysis of the transition of metabolic status included the subgroup of 8521 participants who had complete re-assessment of their metabolic status at median follow-up of 4.4 (IQR 3.7–4.9) years.

### Obesity

Height was measured to the nearest centimetre, using a Seca 202 stadiometer (Hamburg, Germany), and body weight to the nearest 0.1 kg, using a Tanita BC-418 body composition analyser (Tokyo, Japan). BMI was calculated as weight/height^2^. Participants with BMI ≥ 30 kg/m^2^ were classified as obese.

### Metabolic status

Our analyses included six metabolic markers to define metabolic health as a binary condition, including BP and five blood-based biomarkers: C-reactive protein (CRP), triacylglycerols, LDL-cholesterol, HDL-cholesterol and HbA_1c_. BP was measured by a nurse using an automated machine (or manually if unavailable), and the mean of available measurements was derived. Serum biomarkers were measured at a dedicated central laboratory between 2014 and 2017. These measures were externally quality assured and were quality controlled [18]. The cut-off values to define metabolically healthy were adapted from a previous study [19] and are shown in Electronic supplementary material (ESM) Table 1. Participants who fulfilled at least four (out of six) metabolically healthy criteria were considered metabolically healthy.

### Metabolic health and obesity

Based on BMI and metabolic status, participants were categorised as having: metabolically healthy non-obesity (MHN, reference group), MHO, metabolically unhealthy non-obesity (MUN) or MUO. In a sensitivity analysis, MHN and MUN were combined as non-obesity.

### Outcomes

Outcomes were ascertained through individual-level record linkage of the UK Biobank cohort to routine administrative databases. Date and cause of death were obtained from death certificates held by the National Health Service Information Centre (England and Wales) and the National Health Service Central Register (Scotland). Dates and causes of hospital admissions were obtained through record linkage to Health Episode Statistics (England and Wales) and Scottish Morbidity Records (Scotland). Detailed information about the linkage procedures can be found at http://content.digital.nhs.uk/services. Incident diabetes was ascertained through linkage to the primary care data, which are available in about 45% of all participants. Primary care data were used to ascertain diabetes because patients with diabetes are routinely managed in general practitioner practices in the UK. At the time of analysis, mortality data were available up to 30 June 2020, hospital admission data were available up to 31 May 2020 for participants in England and 31 March 2017 for those in Scotland and Wales, and primary care data were available up to 2016 to 2017. We defined diabetes as type 2 diabetes mellitus (ICD-10 code E11), and ASCVD as fatal ischaemic heart disease (I10–25), or non-fatal myocardial infarction (MI) (I21), or fatal/non-fatal stroke (I60–64) per the American College of Cardiology definition [20]. In addition, HF was defined as I11.0, I42.0, I42.6–42.7, I42.9 and I50; chronic respiratory diseases as I26–28 and J30–99; and chronic obstructive pulmonary disease (COPD) as J41–44.

### Covariates

Ethnicity, highest level of education, television viewing time, smoking status, alcohol consumption and dietary intake were self-reported. Physical activity was self-reported using the validated International Physical Activity Questionnaire [21]. Townsend area deprivation index was obtained from postcode of residence and is derived using aggregated data on unemployment, car and home ownership, and household overcrowding [22]. Baseline prevalent conditions were self-reported in a nurse-led interview.

### Statistical analyses

Means and SDs were used to describe continuous variables. Cox proportional hazards models were used to analyse the associations between MHO and health outcomes, with the results reported as HRs and 95% CIs. The models were adjusted for age, sex and ethnicity in Model 1; and additionally for education level and deprivation index in Model 2; and additionally for smoking, alcohol drinking, television viewing, physical activity and intake of fruit and vegetables, oily fish, red meat and processed meat in Model 3. These factors were chosen as they are plausible confounders, based on prior knowledge.

The dose–response relationships of BMI and metabolic status with health outcomes were analysed using a penalised cubic spline in a Cox model, adjusting for the same covariates, with four metabolically healthy criteria as the reference points. Penalised splines are a variation of basis splines and are robust against the number of knots and knot placements [23]. The interactions between BMI and metabolic status were tested by using likelihood ratio tests.

Two sensitivity analyses were conducted. First, non-obesity (whether MHN or MUN) was used as the reference group to examine the risk of MHO compared with all non-obese participants, instead of MHN. Second, we conducted a 5 year landmark analysis, excluding any participant who developed the outcome of interest in the first 5 years of follow-up to mitigate the potential effect of reverse causation.

Participants with longitudinal BMI and metabolic data were categorised as MHN throughout, MHO throughout, transition from MHO to MUO, or MUO throughout, which covers the most common scenarios. These four categories were then used as a primary exposure variable in Cox models. Participants not in any of the four categories were excluded in the transition analysis as there were not sufficient numbers of events to support reliable inference. Proportional hazards assumptions were checked using Schoenfeld residuals.

The mechanistic role of diabetes between MHO and outcomes was explored in two steps. We first examined whether incident diabetes was still a significant predictor of outcomes after adjusting for MHO and other covariates. If so, formal mediation analyses using the counterfactual-based mediation framework were conducted [24]. All analyses were conducted using R version 4.0.3 with the package *survival* (https://www.r-project.org/ accessed 11 October 2020).

### Ethics approval

UK Biobank received ethics approval from the North West Multi-Centre Research Ethics Committee (REC reference: 11/NW/03820). All participants gave written informed consent before enrolment in the study, which was conducted in accordance with the principles of the Declaration of Helsinki. Direct dissemination of the results to participants is not possible/applicable.

## Results

There were 381,363 participants included in the main analysis (Fig. 1), of which 208,625 (54.7%) had MHN, 35,103 (9.2%) MHO, 78,259 (20.5%) MUN and 59,376 (15.6%) MUO. Participants with MUO at baseline, compared with MHN, were older, watched more television, exercised less, had lower education level, higher deprivation index and higher red and processed meat intake, were more likely to be male and of non-white ethnicity, and were less likely to be a current smoker (Table 1). Compared with MUO, people with MHO were younger, watched less television, exercised more, had higher education level, lower deprivation index and higher red and processed meat intake, and were less likely to be male and of non-white ethnicity.

**Table 1:** Participant characteristics

Characteristic	MHN	MHO	MUN	MUO
Total *N*	208,625 (54.71)	35,103 (9.20)	78,259 (20.52)	59,376 (15.57)
Male	84,648 (40.57)	15,166 (43.20)	47,292 (60.43)	30,591 (51.52)
Age, years, mean (SD)	55.80 (8.19)	56.04 (8.11)	58.52 (7.68)	57.53 (7.67)
Ethnicity				
White	198,496 (95.55)	32,817 (93.98)	73,060 (93.79)	55,913 (94.70)
South Asian	1243 (0.60)	230 (0.66)	372 (0.48)	333 (0.56)
Black	3183 (1.53)	454 (1.30)	2617 (3.36)	1147 (1.94)
Chinese	2356 (1.13)	1022 (2.93)	808 (1.04)	1091 (1.85)
Mixed	803 (0.39)	27 (0.08)	286 (0.37)	35 (0.06)
Other	1649 (0.79)	370 (1.06)	753 (0.97)	524 (0.89)
Education level				
College or university degree	77,114 (36.99)	9475 (27.03)	21,896 (28.01)	13,294 (22.42)
A levels/AS levels or equivalent	24,610 (11.81)	3747 (10.69)	7995 (10.23)	5956 (10.04)
O levels/GCSE or equivalent	44,157 (21.18)	7913 (22.57)	16,569 (21.19)	12,870 (21.70)
CSEs or equivalent	10,932 (5.24)	2396 (6.84)	3926 (5.02)	3687 (6.22)
NVQ or HND or HNC or equivalent	11,809 (5.67)	2579 (7.36)	5936 (7.59)	4912 (8.28)
Other professional qualifications	10,216 (4.90)	1895 (5.41)	4268 (5.46)	3410 (5.75)
Prefer not to answer	1813 (0.87)	434 (1.24)	1037 (1.33)	852 (1.44)
None of the above	27,795 (13.33)	6615 (18.87)	16,558 (21.18)	14,326 (24.16)
Deprivation index, mean (SD)	−1.55 (2.94)	−1.03 (3.17)	−1.32 (3.09)	−0.78 (3.26)
Smoking				
Never	120,690 (58.10)	18,931 (54.24)	38,372 (49.29)	28,746 (48.74)
Previous	67,773 (32.63)	13,183 (37.77)	28,197 (36.22)	23,944 (40.60)
Current	19,257 (9.27)	2787 (7.99)	11,285 (14.50)	6292 (10.67)
Alcohol consumption, units/week, mean (SD)	15.81 (17.24)	16.78 (20.17)	17.94 (20.78)	16.45 (21.26)
TV viewing, hours/day, mean (SD)	2.54 (1.46)	3.05 (1.58)	3.00 (1.61)	3.39 (1.74)
Physical activity, MET min/week, mean (SD)	2773.06 (2249.72)	2530.02 (2166.41)	2629.98 (2212.36)	2362.97 (2073.92)
Fruit/vegetable intake, portions/week, mean (SD)	4.21 (2.41)	4.16 (2.52)	3.91 (2.45)	4.00 (2.47)
Oily fish intake				
Never	20,250 (9.76)	3972 (11.40)	9197 (11.85)	7750 (13.18)
Less than once a week	67,696 (32.63)	11,419 (32.79)	26,165 (33.71)	20,092 (34.17)
Once a week	80,672 (38.89)	12,841 (36.87)	28,952 (37.30)	21,102 (35.89)
2–4 times a week	36,758 (17.72)	6222 (17.87)	12,632 (16.28)	9356 (15.91)
5–6 times a week	1616 (0.78)	271 (0.78)	497 (0.64)	344 (0.59)
Once or more daily	471 (0.23)	102 (0.29)	170 (0.22)	153 (0.26)
Red meat intake, portions/week, mean (SD)	2.00 (1.38)	2.21 (1.50)	2.18 (1.48)	2.32 (1.57)
Processed meat intake				
Never	22,931 (11.02)	2431 (6.95)	5898 (7.56)	3268 (5.53)
Less than once a week	68,018 (32.70)	10,355 (29.62)	21,258 (27.26)	15,105 (25.55)
Once a week	59,829 (28.76)	10,387 (29.71)	23,265 (29.84)	17,767 (30.06)
2–4 times a week	50,111 (24.09)	10,360 (29.64)	23,875 (30.62)	19,898 (33.66)
5–6 times a week	5753 (2.77)	1130 (3.23)	2884 (3.70)	2425 (4.10)
Once or more daily	1375 (0.66)	293 (0.84)	790 (1.01)	647 (1.09)
BMI, kg/m^2^, mean (SD)	24.94 (2.63)	33.05 (3.04)	26.62 (2.24)	34.42 (4.03)
Number of MH factors, mean (SD)	4.47 (0.64)	4.22 (0.46)	2.67 (0.58)	2.41 (0.77)
SBP, mmHg, mean (SD)	134.17 (18.59)	138.62 (18.28)	144.03 (16.92)	144.04 (17.16)
DBP, mmHg, mean (SD)	79.87 (9.84)	84.38 (9.85)	84.58 (9.36)	86.67 (9.61)
Any antihypertensive medications	26,858 (12.87)	9164 (26.11)	22,997 (29.39)	25,255 (42.53)
CRP, mg/l, mean (SD)	1.45 (3.15)	2.37 (3.59)	4.12 (6.39)	5.18 (5.91)
Triacylglycerols, mmol/l, mean (SD)	1.32 (0.56)	1.51 (0.57)	2.46 (1.20)	2.51 (1.25)
LDL-cholesterol, mmol/l, mean (SD)	3.50 (0.82)	3.46 (0.86)	3.73 (0.93)	3.58 (0.93)
HDL-cholesterol, mmol/l, mean (SD)	1.58 (0.37)	1.40 (0.30)	1.29 (0.35)	1.21 (0.30)
Any cholesterol lowering medications	22,495 (10.78)	6560 (18.69)	20,104 (25.69)	19,805 (33.36)
HbA_1c_, mmol/mol, mean (SD)	34.42 (3.93)	35.35 (4.23)	37.90 (8.52)	40.45 (10.34)
HbA_1c_, %, mean	5.3	5.4	5.6	5.9

Numbers are *n* (%) unless otherwise specified

Some sub-categories, such as education level, may not add up due to missing data

A levels, Advanced levels; AS levels, Advanced Subsidiary levels; CRP, C-reactive protein; CSEs, Certificate of Secondary Education; DBP, diastolic BP; GCSE, General Certificate of Secondary Education; HNC, Higher National Certificates; HND, Higher National Diplomas; MET, metabolic equivalent of tasks; MH, metabolically healthy; NVQ, National Vocational Qualification; O levels, Ordinary levels; SBP, systolic BP; TV, television

**Fig. 2:**
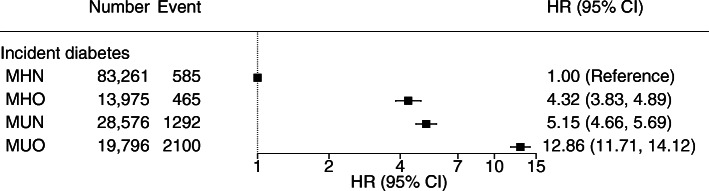
Association between MHO and incident diabetes with MHN as reference group. Adjusted for each other and for age, sex, ethnicity, education, deprivation, smoking, alcohol drinking, television viewing, physical activity and intake of fruit and vegetables, oily fish, red meat and processed meat

**Fig. 3:**
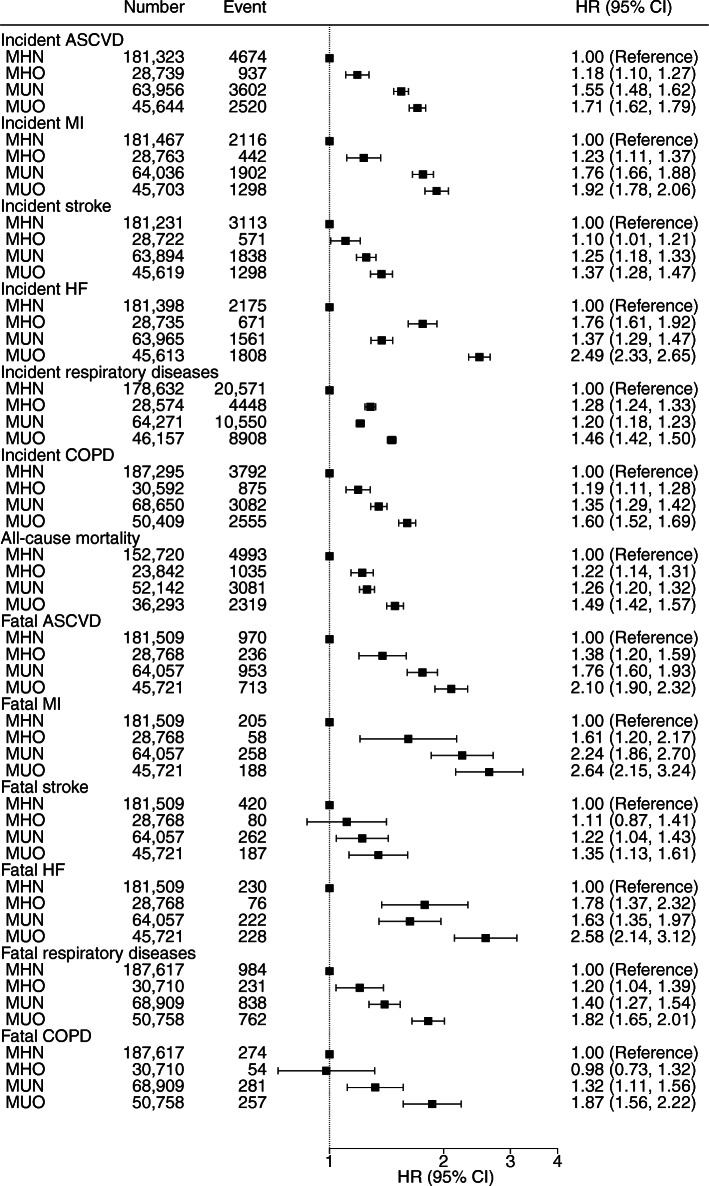
Association between MHO and cardiovascular and respiratory outcomes with MHN as reference group. Adjusted for each other and for age, sex, ethnicity, education, deprivation, smoking, alcohol drinking, television viewing, physical activity, and intake of fruit and vegetables, oily fish, red meat and processed meat

The median (IQR) follow-up period was 11.2 (10.3–11.9) years. In Model 3, compared with participants with MHN at baseline, those with MHO had higher rates of incident diabetes (HR 4.32; 95% CI 3.83, 4.89) (Fig. 2), ASCVD (HR 1.18; 95% CI 1.10, 1.27), MI (HR 1.23; 95% CI 1.11, 1.37), stroke (HR 1.10; 95% CI 1.01, 1.21), HF (HR 1.76; 95% CI 1.61, 1.92), respiratory diseases (HR 1.28; 95% CI 1.24, 1.33) and COPD (HR 1.19; 95% CI 1.11, 1.28) (Fig. 3). Generally, rates of cardiovascular and respiratory outcomes were highest in MUO, followed by MUN and MHO, except for incident and fatal HF, and incident respiratory diseases. In these outcomes, people with MHO had higher rates than those with MUN. The associations between MHO and mortality outcomes were generally similar, except that MHO was not significantly associated with stroke or COPD mortality. Participants with MHO had higher all-cause mortality rates (HR 1.22; 95% CI 1.14, 1.31) compared with participants with MHN.

Compared with participants without obesity (regardless of MHN or MUN) at baseline, those with MHO had higher rates of diabetes (HR 2.06; 95% CI 1.77, 2.40), HF (HR 1.60; 95% CI 1.45, 1.75) and respiratory diseases (HR 1.20; 95% CI 1.16, 1.25), but not ASCVD, including MI and stroke (ESM Fig. 1). The associations of MHO were generally weaker for fatal outcomes and only significant for all-cause mortality (HR 1.12; 95% CI 1.04, 1.21) and HF mortality rates (HR 1.44; 95% CI 1.09, 1.89). The results for Models 1 and 2 are shown in ESM Tables 2 and 3. The results of the 5 year landmark analysis were similar (ESM Fig. 2).

The interactions between obesity and metabolic health factors are shown in ESM Fig. 3. The associations of metabolic health factors with incident respiratory diseases (*p*_interaction_ = 0.001) were slightly stronger among people who were not obese. There were no other significant interactions. There were no significant time trends in the Schoenfeld residuals, except for diabetes, suggesting these associations were relatively stable during the follow-up (ESM Fig. 4). The trend for diabetes appeared to be increasing, suggesting the association between MHO and diabetes may be increasing over time, and the current HR estimate should not be extrapolated for different follow-up duration.

**Fig. 4:**
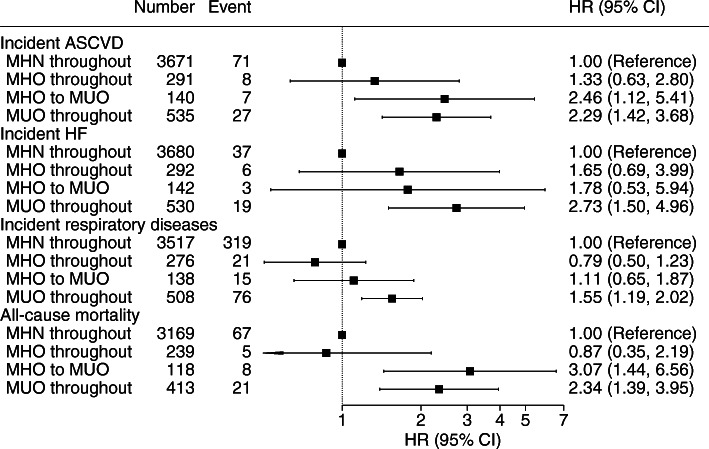
Association of change of metabolic status with health outcomes. Adjusted for age, sex ethnicity, education, deprivation, smoking, alcohol drinking, television viewing, physical activity, and intake of fruit and vegetables, oily fish, red meat and processed meat

ESM Fig. 5 shows the transition of metabolic status among the subgroup of 8521 participants who had longitudinal BMI and metabolic data (median [IQR] follow-up 4.4 [3.7–4.9] years). Half of the participants who had MHO at baseline remained so in the follow-up, 20% became non-obese and over one-quarter transitioned to MUO. Figure 4 shows the associations between transition of MHO status and health outcomes. Compared with participants with MHN throughout, participants who transitioned from MHO to MUO had higher rates of incident ASCVD (HR 2.46; 95% CI 1.12, 5.41) and all-cause mortality (HR 3.07; 95% CI 1.44, 6.56). There were no significant associations for the MHO-throughout group. After controlling for baseline MHO status, incident diabetes was no longer a significant risk factor for any of the outcomes (ESM Table 4) and thus was not a mediator.

## Discussion

### Principal findings

The current study demonstrated that people with MHO were at a substantially higher risk of diabetes, ASCVD, HF, respiratory diseases and all-cause mortality compared with people with MHN. Particularly worth noting is that people with MHO were at an even higher risk than those with MUN of HF and respiratory disease. Among people with MHO at baseline who remained obese, over one-third became metabolically unhealthy within 3 to 5 years. These people acquired an even higher risk of ASCVD. In addition, there were weak or non-significant interactions of obesity and metabolic health factors with health outcomes. The key point therefore is that the risk of many important outcomes, such as HF and respiratory disease, is elevated in people with obesity even if they have a normal metabolic profile. Using the label ‘metabolically healthy’ to describe this group in clinical medicine is misleading and therefore should be avoided.

### Strengths and limitations of this study

This study has several strengths over the previous evidence. First, we were able to investigate emerging outcomes such as HF and respiratory diseases that have often been omitted in MHO studies. In fact, the associations with these outcomes, along with the instability of the MHO status, highlight that MHO is not a healthy state. Furthermore, this study conducted a 5 year landmark analysis which indicated that the findings were unlikely to simply reflect reverse causation. We also adjusted for a wide range of potential confounders, including individual- and area-level socioeconomic status and lifestyle factors. Using mediation analysis, the current study also found that individuals with MHO may develop ASCVD and HF outcomes through diabetes-independent pathways. However, as with any observational study, residual confounding, such as from excess energy intake and family history of diabetes and ASCVD, is possible. Furthermore, whilst the UK Biobank cohort broadly reflects the general population in terms of sociodemographic characteristics, it is not representative in terms of lifestyle [25]. Therefore, whilst relative risk should be generalisable, as shown in a previous analysis [26], summary statistics and estimates of absolute risk should not be generalised. In addition, fasting glucose and insulin resistance were not measured, which limited how well we could define metabolically healthy states. However, HbA_1c_ is a well-established measure to reflect the mean blood glucose level in the last 2–3 months, and predicts relevant outcomes with similar efficacy to other blood glucose level measures [27]. This study also did not consider alternative definitions of MHO, e.g. by using the absence of hospitalisation [28, 29], or by using other genetics and omics data, which could be explored in future studies. Factors associated with MHO transitioning to MUO (or lack thereof) could also be explored.

### Strengths and limitations compared with other studies

Some of the results of our study are consistent with the results of previous studies, lending external validity. In a study of 5269 adults aged 39–62 years followed up for 17.7 years, MHO was associated with high risk of all-cause mortality (HR 1.81, 95% CI 1.16 to 2.84) compared with MHN [7]. The same results were found in a prospective study of 1758 middle-aged men from Sweden followed up over 30 years [12]. The long-term relationship between MHO and CVD has been explored in several existing studies [7, 12]. Consistent with our findings, a multi-national European study found those with MHO to have a higher CVD risk than MHN but lower than MUN and MUO [30]. Conversely, there have been studies showing no association between MHO and CVD, such as a UK study that followed 22,203 participants over 12.7 years [11]. However, the effect size reported in that study was similar to our current study, suggesting that their lack of statistical significance could be due to insufficient power. It is worth noting that this previous literature has mostly focused on ASCVD, including ischaemic heart disease and stroke, and often omitted HF. Importantly, we found that MHO was associated with substantially higher risk of HF even compared with those with MUN. This could be related to a range of mechanistic factors beyond the usual metabolic aspect, such as haemodynamic perturbances, that likely link obesity to HF risk [31]. Notably, others have shown that obesity may be more strongly linked to incident HF than to MI [32].

To our knowledge, no previous study has directly examined the association between MHO and respiratory diseases, including COPD. However, it is well recognised that obesity is generally associated with lower respiratory function and a wide range of respiratory diseases, such as COPD, obstructive sleep apnoea and obesity hypoventilation syndrome [33]. There have been several hypotheses as to why obesity may be associated with COPD, including fat oxidative capacity, inflammation and insulin resistance [34]. However, as shown in this study, having a normal metabolic profile, and HbA_1c_, did not guarantee lower risk of respiratory diseases among obese people. Of note, chronic respiratory diseases account for considerable morbidity and mortality worldwide and are estimated to have been the third leading cause of death in 2017 [35].

### Implications of this study

Although our results suggested that people with MHO may have lower cardiovascular and respiratory risk compared with MUO, their risk was still higher than those who were metabolically healthy without obesity, especially with respect to HF and respiratory diseases. These findings, as well as the unstable nature of MHO, suggest that weight management could be beneficial for people with obesity even if they do not currently show abnormalities in their metabolic profile. Weight management strategies include lifestyle changes, such as diet and physical activity, concomitant pharmacotherapies upon risk assessment [36, 37] or bariatric surgery in severe obesity [38].

It is worth noting that half of the participants remained with MHO after 4.4 years of follow-up. We could not detect any significant elevated risk among them compared with people who were metabolically healthy and non-obese throughout the study. It is likely that this group of people are at lower risk than people with other MHO trajectories. However, since there were not sufficient numbers of events, we cannot conclude whether they were at the same risk as people with MHN, or were at a modestly elevated risk. Future prospective studies should consider this research question.

This study also showed that people with obesity are a heterogenous group, and there is a potential to risk stratify based on prognosis. For example, people with MUO were at a higher risk of mortality and morbidity than everyone else, and thus they should be prioritised for intervention. However, it should be noted that using a single binary label (i.e. ‘MHO’) for clinical management may have questionable utility. Obesity is associated with a wide range of diseases and using a single label (or categorical risk algorithm) is unlikely to be effective compared with prediction algorithms based on disease-specific and continuous risk markers. It has been shown that the metabolic syndrome, a similar categorical risk criterion to MHO, predicted neither ASCVD nor diabetes satisfactorily [39].

### Conclusions

People with MHO are not ‘healthy’ as they are at higher risk of ASCVD, HF and respiratory diseases compared with non-obese people with a normal metabolic profile. As such, weight management could be beneficial to all people with obesity irrespective of metabolic profile. We suggest the term ‘MHO’ should be avoided in clinical medicine as it is misleading, and different strategies for risk stratification should be explored.

## Supplementary Information

ESM (PDF 1198 kb)

## Data Availability

UK Biobank data can be requested by bona fide researchers for approved projects, including replication, through https://www.ukbiobank.ac.uk/.

## Abbreviations

ASCVD: Atherosclerotic CVD
COPD: Chronic obstructive pulmonary disease
HF: Heart failure
MHN: Metabolically healthy non-obesity
MHO: Metabolically healthy obesity
MI: Myocardial infarction
MUN: Metabolically unhealthy non-obesity
MUO: Metabolically unhealthy obesity
