# Supplementary material for: Are people with metabolically healthy obesity really healthy? A prospective cohort study of 381,363 UK Biobank participants
Source: Diabetologia. 2021 Jun 10;64(9):1963–72. doi: 10.1007/s00125-021-05484-6 (PMC8382657; doi:10.1007/s00125-021-05484-6)

Electronic Supplementary Materials

Are people with metabolically healthy obesity really healthy? A prospective cohort study of 381,363 UK Biobank participants

Ziyi Zhou[1], John Macpherson[1], Stuart R Gray[2], Jason M R Gill[2], Paul Welsh[2], Carlos Celis-Morales[1,2,3], Naveed Sattar[2], Jill P Pell[1], Frederick K Ho[1]

[1]Institute of Health and Wellbeing, University of Glasgow, Glasgow, UK
[2]Institute of Cardiovascular and Medical Sciences, University of Glasgow, Glasgow, UK
[3]Human Performance Lab, Education, Physical Activity and Health Research Unit, University Católica del Maule, Talca, Chile

Carlos Celis-Morales, Naveed Sattar, Jill P. Pell and Frederick K. Ho are joint senior authors.

Corresponding author:
Frederick Ho,
Email: Frederick.Ho@glasgow.ac.uk

ESM Table 1. Criteria for metabolically healthy

**Biomarker**	**Criteria for Metabolically Healthy**
BP	SBP <130 mmHg and DBP <80 mmHg and no antihypertensive medications
C-reactive protein (CRP)	CRP <3 mg/L
Triacylglycerols	Triacylglycerols <2.3 mmol/L
Low-density lipoprotein cholesterol (LDL-c)	LDL-c <3 mmol/L and no cholesterol lowering medications
High-density lipoprotein cholesterol (HDL-c)	HDL-c >1 mmol/L
Glycated haemoglobin	HbA_{1c} <42 mmol/mol (6%) and no diabetes medications

ESM Table 2. Association between MHO and incidence outcomes

	Model 1		Model 2	
	**HR (95% CI)**	**P**	**HR (95% CI)**	**P**
**Diabetes**				
MHN	1 (Reference)		1 (Reference)	
MHO	4.79 (4.27, 5.37)	<0.0001	4.55 (4.05, 5.10)	<0.0001
MUN	5.67 (5.16, 6.22)	<0.0001	5.43 (4.94, 5.96)	<0.0001
MUO	14.79 (13.56, 16.13)	<0.0001	13.72 (12.57, 14.98)	<0.0001
**ASCVD**				
MHN	1 (Reference)		1 (Reference)	
MHO	1.22 (1.14, 1.30)	<0.0001	1.15 (1.08, 1.23)	<0.0001
MUN	1.68 (1.61, 1.75)	<0.0001	1.62 (1.55, 1.69)	<0.0001
MUO	1.90 (1.82, 1.99)	<0.0001	1.77 (1.69, 1.85)	<0.0001
**MI**				
MHN	1 (Reference)		1 (Reference)	
MHO	1.26 (1.14, 1.39)	<0.0001	1.20 (1.09, 1.32)	0.0003
MUN	1.93 (1.82, 2.05)	<0.0001	1.87 (1.76, 1.98)	<0.0001
MUO	2.14 (2.00, 2.28)	<0.0001	1.99 (1.87, 2.13)	<0.0001
**Stroke**				
MHN	1 (Reference)		1 (Reference)	
MHO	1.12 (1.03, 1.22)	0.01	1.07 (0.98, 1.17)	0.12
MUN	1.32 (1.25, 1.39)	<0.0001	1.28 (1.21, 1.36)	<0.0001
MUO	1.48 (1.39, 1.57)	<0.0001	1.40 (1.31, 1.49)	<0.0001
**HF**				
MHN	1 (Reference)		1 (Reference)	
MHO	1.93 (1.78, 2.10)	<0.0001	1.81 (1.67, 1.96)	<0.0001
MUN	1.55 (1.46, 1.65)	<0.0001	1.49 (1.40, 1.58)	<0.0001
MUO	2.93 (2.76, 3.11)	<0.0001	2.68 (2.52, 2.84)	<0.0001
**Respiratory disease**				
MHN	1 (Reference)		1 (Reference)	
MHO	1.37 (1.33, 1.41)	<0.0001	1.30 (1.26, 1.35)	<0.0001
MUN	1.32 (1.29, 1.35)	<0.0001	1.27 (1.24, 1.30)	<0.0001
MUO	1.65 (1.61, 1.68)	<0.0001	1.53 (1.49, 1.57)	<0.0001
**COPD**				
MHN	1 (Reference)		1 (Reference)	
MHO	1.41 (1.31, 1.51)	<0.0001	1.18 (1.10, 1.27)	<0.0001
MUN	1.82 (1.74, 1.90)	<0.0001	1.62 (1.55, 1.70)	<0.0001
MUO	2.24 (2.13, 2.35)	<0.0001	1.74 (1.66, 1.83)	<0.0001

Model 1: Adjusted for age, sex, and ethnicity only.
Model 2: Additionally adjusted for education level and deprivation index.
MHN: metabolically healthy non-obese; MHO; metabolically healthy obese; MUN; metabolically unhealthy non-obese; MUO metabolically unhealthy obese

ESM Table 3. Association between MHO and mortality outcomes

	Model 1		Model 2	
	**HR (95% CI)**	**P**	**HR (95% CI)**	**P**
**All-cause mortality**				
MHN	1 (Reference)		1 (Reference)	
MHO	1.27 (1.19, 1.36)	< 0.0001	1.21 (1.13, 1.29)	< 0.0001
MUN	1.38 (1.33, 1.44)	< 0.0001	1.34 (1.28, 1.40)	< 0.0001
MUO	1.69 (1.62, 1.77)	< 0.0001	1.57 (1.49, 1.64)	< 0.0001
**ASCVD**				
MHN	1 (Reference)		1 (Reference)	
MHO	1.47 (1.28, 1.68)	< 0.0001	1.36 (1.18, 1.56)	< 0.0001
MUN	1.96 (1.80, 2.14)	< 0.0001	1.87 (1.72, 2.04)	< 0.0001
MUO	2.50 (2.29, 2.74)	< 0.0001	2.23 (2.04, 2.45)	< 0.0001
**MI**				
MHN	1 (Reference)		1 (Reference)	
MHO	1.76 (1.34, 2.33)	< 0.0001	1.63 (1.23, 2.15)	0.0006
MUN	2.53 (2.12, 3.02)	< 0.0001	2.41 (2.02, 2.88)	< 0.0001
MUO	3.08 (2.55, 3.71)	< 0.0001	2.74 (2.27, 3.32)	< 0.0001
**Stroke**				
MHN	1 (Reference)		1 (Reference)	
MHO	1.13 (0.90, 1.43)	0.30	1.06 (0.84, 1.34)	0.63
MUN	1.32 (1.14, 1.53)	0.0003	1.27 (1.09, 1.47)	0.002
MUO	1.59 (1.36, 1.87)	< 0.0001	1.45 (1.23, 1.71)	< 0.0001
**HF**				
MHN	1 (Reference)		1 (Reference)	
MHO	2.11 (1.65, 2.70)	< 0.0001	1.92 (1.50, 2.46)	< 0.0001
MUN	2.01 (1.68, 2.40)	< 0.0001	1.90 (1.59, 2.27)	< 0.0001
MUO	3.40 (2.85, 4.05)	< 0.0001	2.97 (2.49, 3.55)	< 0.0001
**Respiratory disease**				
MHN	1 (Reference)		1 (Reference)	
MHO	1.40 (1.23, 1.60)	< 0.0001	1.24 (1.09, 1.42)	0.002
MUN	1.72 (1.57, 1.87)	< 0.0001	1.60 (1.46, 1.74)	< 0.0001
MUO	2.38 (2.18, 2.60)	< 0.0001	2.00 (1.83, 2.19)	< 0.0001
**COPD**				
MHN	1 (Reference)		1 (Reference)	
MHO	1.09 (0.83, 1.44)	0.53	0.86 (0.65, 1.13)	0.27
MUN	1.96 (1.68, 2.29)	< 0.0001	1.68 (1.44, 1.97)	< 0.0001
MUO	2.71 (2.31, 3.18)	< 0.0001	1.92 (1.63, 2.26)	< 0.0001

Model 1: Adjusted for age, sex, and ethnicity only.
Model 2: Additionally adjusted for education level and deprivation index.
MHN: metabolically healthy non-obese; MHO; metabolically healthy obese; MUN; metabolically unhealthy non-obese; MUO metabolically unhealthy obese

ESM Table 4. Association between incident diabetes and subsequent outcomes with adjustment of baseline MHO status

	**HR (95% CI)**	**P**
Incident ASCVD	0.68 (0.59, 0.80)	<0.0001
Incident HF	1.00 (0.86, 1.17)	0.97
All-cause mortality	0.88 (0.79, 0.99)	0.03
Fatal ASCVD	0.83 (0.66, 1.06)	0.14
Fatal HF	1.29 (0.89, 1.87)	0.18

Adjusted for age, sex ethnicity, education, deprivation, smoking, alcohol drinking, television viewing, physical activity, intake of fruit and vegetables, oily fish, red meat and processed meat, and baseline MHO status

Incident diabetes was the exposure variable coded as 1 when diabetes was recorded between the baseline assessment and the incidence of the outcome (for people who developed the outcome) or the date of censoring (for censored observations), or 0 otherwise.

ESM Figure 1. Association between MHO and health outcomes with non-obesity as reference group

	Number	Event	HR (95% CI)
Incident diabetes			
Non-obesity	110,791	831	1.00 (Reference)
MHO	13,727	217	2.06 (1.77, 2.40)
MUO	18,473	777	4.77 (4.32, 5.28)
Incident ASCVD			
Non-obesity	242,511	5508	1.00 (Reference)
MHO	28,427	625	1.00 (0.92, 1.09)
MUO	44,777	1653	1.41 (1.34, 1.50)
Incident MI			
Non-obesity	244,042	2557	1.00 (Reference)
MHO	28,611	290	1.00 (0.89, 1.13)
MUO	45,221	816	1.50 (1.38, 1.63)
Incident stroke			
Non-obesity	243,526	3352	1.00 (Reference)
MHO	28,524	373	0.98 (0.88, 1.10)
MUO	45,173	852	1.23 (1.14, 1.33)
Incident HF			
Non-obesity	244,462	2835	1.00 (Reference)
MHO	28,583	519	1.60 (1.45, 1.75)
MUO	45,142	1337	2.16 (2.02, 2.31)
Incident respiratory diseases			
Non-obesity	231,204	19,422	1.00 (Reference)
MHO	26,839	2713	1.20 (1.16, 1.25)
MUO	42,526	5277	1.33 (1.29, 1.38)
Incident COPD			
Non-obesity	254,057	4986	1.00 (Reference)
MHO	30,360	643	1.08 (0.99, 1.17)
MUO	49,577	1723	1.34 (1.27, 1.42)
All-cause mortality			
Non-obesity	202,825	6037	1.00 (Reference)
MHO	23,575	768	1.12 (1.04, 1.21)
MUO	35,754	1780	1.41 (1.33, 1.49)
Fatal ASCVD			
Non-obesity	245,070	1427	1.00 (Reference)
MHO	28,708	176	1.09 (0.93, 1.28)
MUO	45,553	545	1.69 (1.52, 1.87)
Fatal MI			
Non-obesity	245,422	319	1.00 (Reference)
MHO	28,752	42	1.18 (0.85, 1.63)
MUO	45,676	143	2.02 (1.65, 2.48)
Fatal stroke			
Non-obesity	245,419	535	1.00 (Reference)
MHO	28,746	58	0.97 (0.74, 1.28)
MUO	45,678	144	1.26 (1.04, 1.52)
Fatal HF			
Non-obesity	245,472	358	1.00 (Reference)
MHO	28,752	60	1.44 (1.09, 1.89)
MUO	45,688	195	2.23 (1.86, 2.67)
Fatal respiratory diseases			
Non-obesity	256,138	1434	1.00 (Reference)
MHO	30,662	183	1.06 (0.91, 1.24)
MUO	50,597	601	1.60 (1.45, 1.76)
Fatal COPD			
Non-obesity	256,446	475	1.00 (Reference)
MHO	30,704	48	0.89 (0.66, 1.20)
MUO	50,717	216	1.61 (1.36, 1.90)

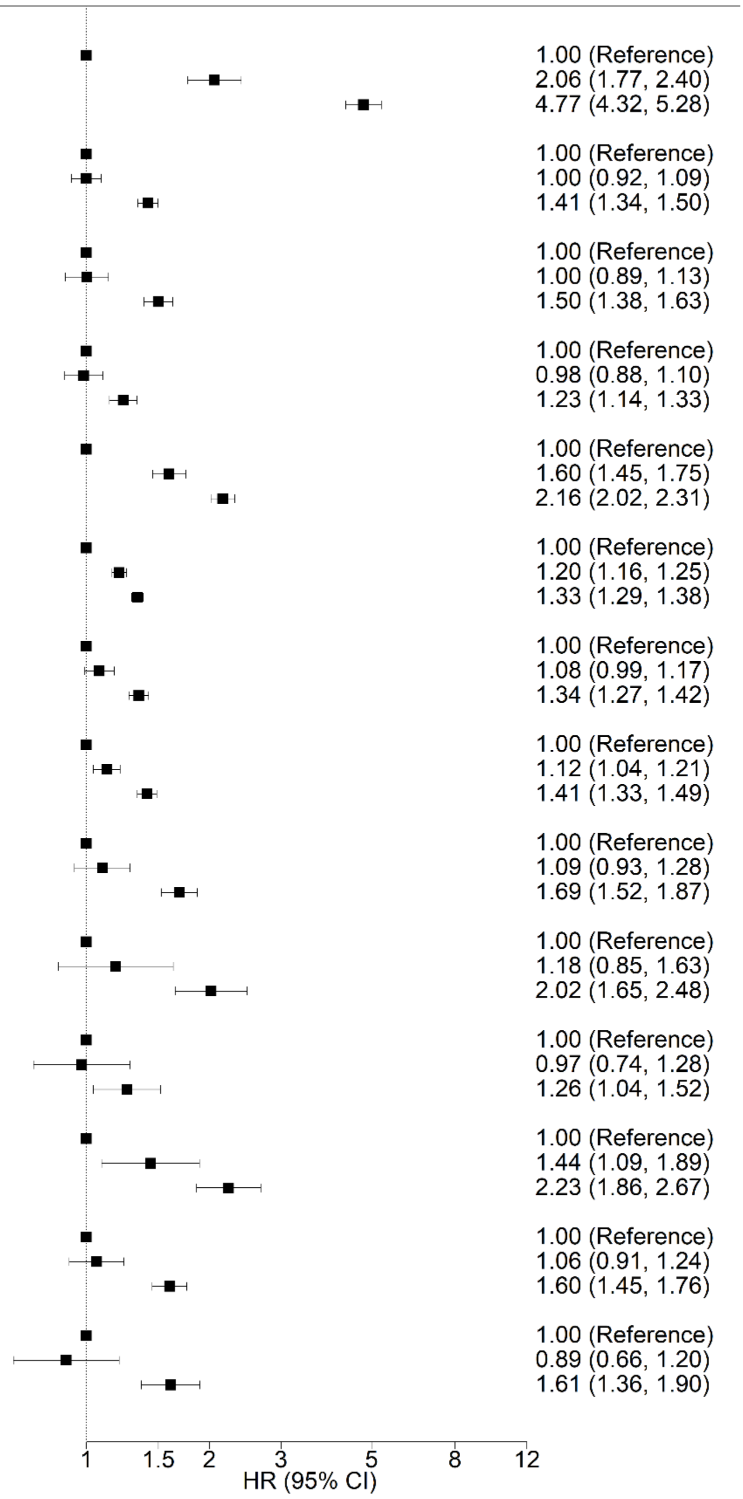

Adjusted for age, sex ethnicity, education, deprivation, smoking, alcohol drinking, television viewing, physical activity, and intake of fruit and vegetables, oily fish, red meat and processed meat
HR shown in logarithmic scale.

ESM Figure 2. Association between MHO and health outcomes in a 5-year landmark analysis

	Number	Event	HR (95% CI)
Incident diabetes			
MHN	82,966	290	1.00 (Reference)
MHO	13,727	217	4.13 (3.46, 4.93)
MUN	27,825	541	4.35 (3.76, 5.03)
MUO	18,473	777	9.83 (8.56, 11.28)
Incident ASCVD			
MHN	179,827	3178	1.00 (Reference)
MHO	28,427	625	1.16 (1.07, 1.27)
MUN	62,684	2330	1.50 (1.42, 1.58)
MUO	44,777	1653	1.67 (1.57, 1.77)
Incident MI			
MHN	180,740	1389	1.00 (Reference)
MHO	28,611	290	1.24 (1.09, 1.41)
MUN	63,302	1168	1.70 (1.57, 1.84)
MUO	45,221	816	1.88 (1.72, 2.05)
Incident stroke			
MHN	180,252	2134	1.00 (Reference)
MHO	28,524	373	1.05 (0.94, 1.18)
MUN	63,274	1218	1.22 (1.13, 1.31)
MUO	45,173	852	1.32 (1.22, 1.44)
Incident HF			
MHN	180,858	1635	1.00 (Reference)
MHO	28,583	519	1.83 (1.65, 2.02)
MUN	63,604	1200	1.42 (1.32, 1.53)
MUO	45,142	1337	2.49 (2.31, 2.68)
Incident respiratory diseases			
MHN	171,013	12,952	1.00 (Reference)
MHO	26,839	2713	1.27 (1.22, 1.32)
MUN	60,191	6470	1.17 (1.14, 1.21)
MUO	42,526	5277	1.41 (1.36, 1.46)
Incident COPD			
MHN	186,307	2804	1.00 (Reference)
MHO	30,360	643	1.20 (1.10, 1.31)
MUN	67,750	2182	1.32 (1.25, 1.40)
MUO	49,577	1723	1.51 (1.42, 1.61)
All-cause mortality			
MHN	151,502	3775	1.00 (Reference)
MHO	23,575	768	1.20 (1.11, 1.30)
MUN	51,323	2262	1.22 (1.16, 1.29)
MUO	35,754	1780	1.52 (1.43, 1.61)
Fatal ASCVD			
MHN	181,264	725	1.00 (Reference)
MHO	28,708	176	1.39 (1.18, 1.64)
MUN	63,806	702	1.74 (1.57, 1.94)
MUO	45,553	545	2.17 (1.93, 2.43)
Fatal MI			
MHN	181,449	145	1.00 (Reference)
MHO	28,752	42	1.68 (1.19, 2.38)
MUN	63,973	174	2.19 (1.75, 2.74)
MUO	45,676	143	2.93 (2.31, 3.72)
Fatal stroke			
MHN	181,417	328	1.00 (Reference)
MHO	28,746	58	1.05 (0.80, 1.40)
MUN	64,002	207	1.24 (1.04, 1.48)
MUO	45,678	144	1.37 (1.12, 1.67)
Fatal HF			
MHN	181,454	175	1.00 (Reference)
MHO	28,752	60	1.85 (1.38, 2.49)
MUN	64,018	183	1.78 (1.44, 2.19)
MUO	45,688	195	2.91 (2.35, 3.59)
Fatal respiratory diseases			
MHN	187,399	766	1.00 (Reference)
MHO	30,662	183	1.24 (1.05, 1.46)
MUN	68,739	668	1.44 (1.30, 1.60)
MUO	50,597	601	1.88 (1.68, 2.10)
Fatal COPD			
MHN	187,574	231	1.00 (Reference)
MHO	30,704	48	1.04 (0.76, 1.42)
MUN	68,872	244	1.37 (1.14, 1.65)
MUO	50,717	216	1.88 (1.55, 2.28)

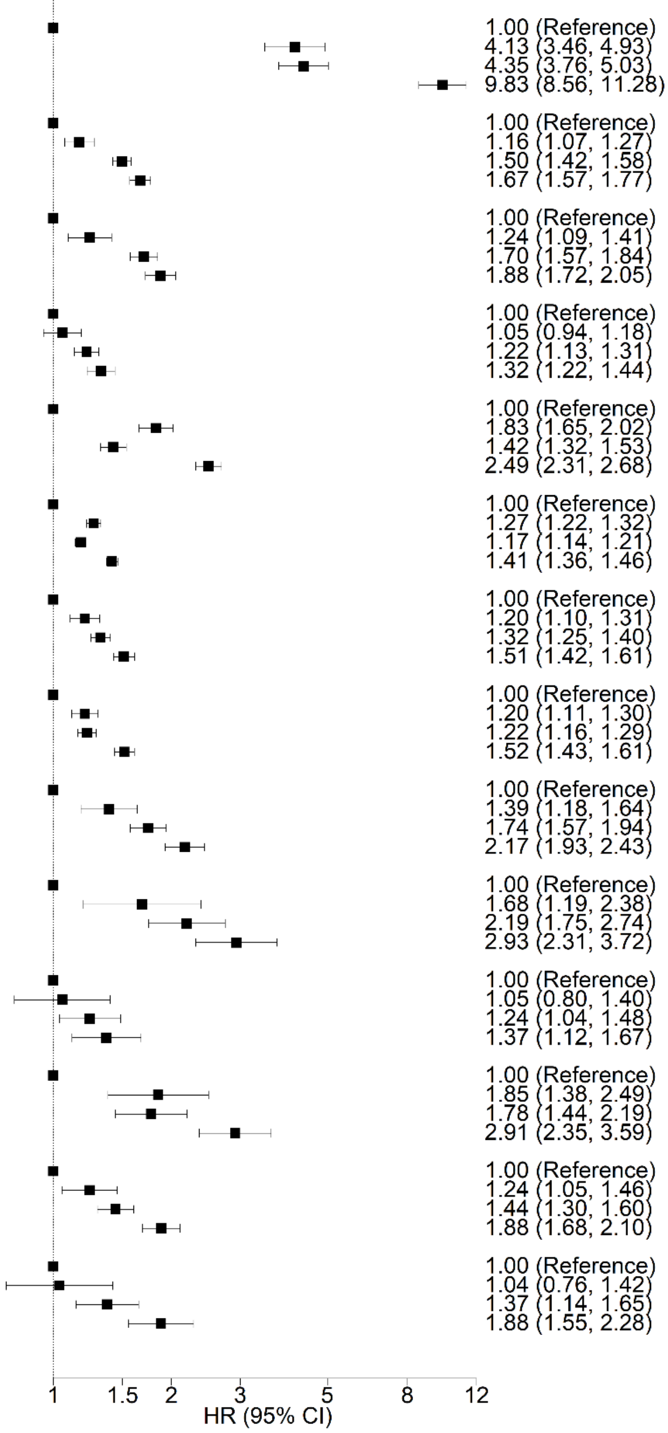

Participants who had corresponding events in the first five years of follow-up were excluded. Adjusted for age, sex ethnicity, education, deprivation, smoking, alcohol drinking, television viewing, physical activity, and intake of fruit and vegetables, oily fish, red meat and processed meat
HR shown in logarithmic scale.

ESM Figure 3. Interaction of obesity and metabolic health with health outcomes

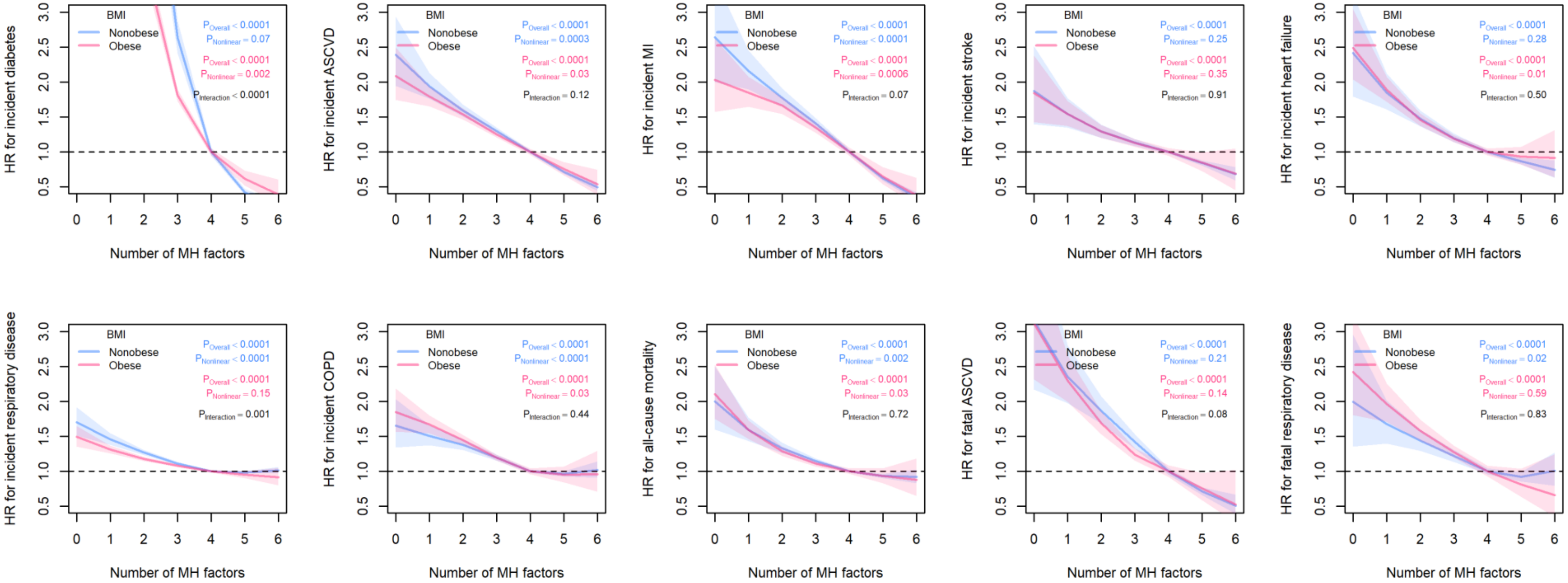

Main effects of obesity were not shown. Adjusted for each other and for age, sex ethnicity, education, deprivation, smoking, alcohol drinking, television viewing, physical activity, and intake of fruit and vegetables, oily fish, red meat and processed meat.

ESM Figure 4. Schoenfeld residuals for MHO

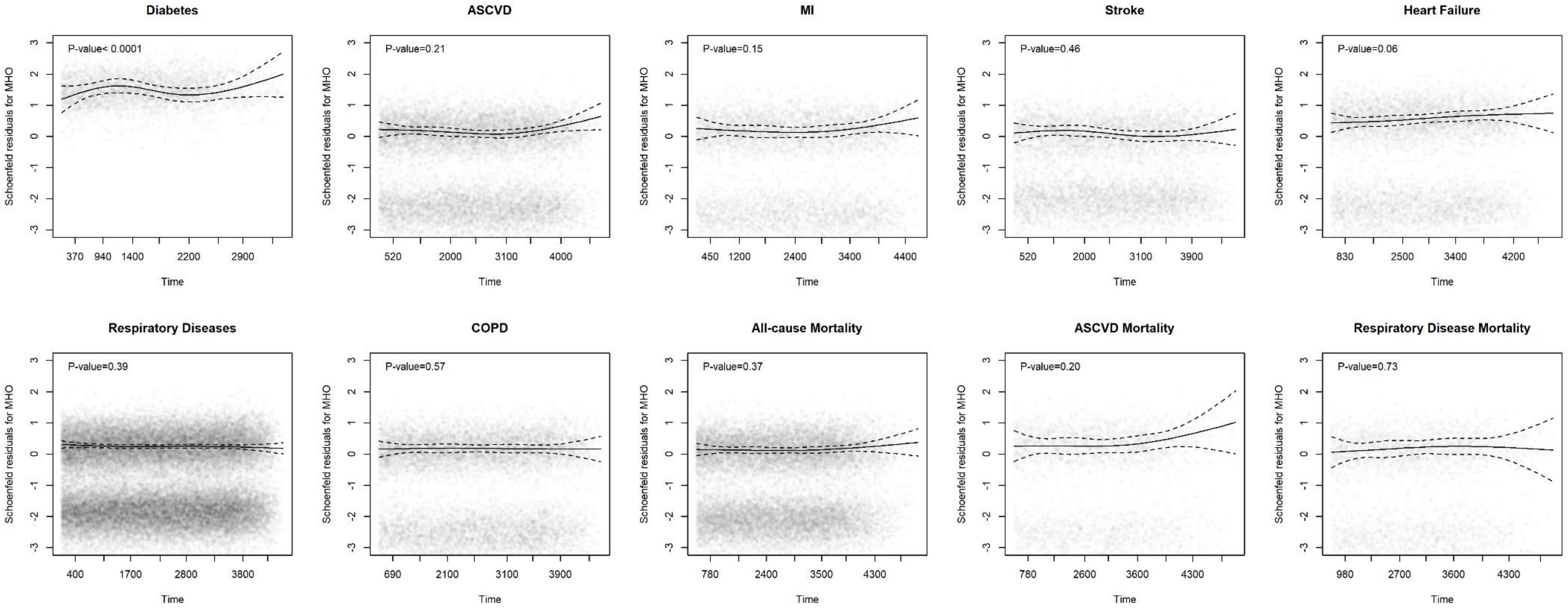

ESM Figure 5. Transition of metabolic health and obesity status

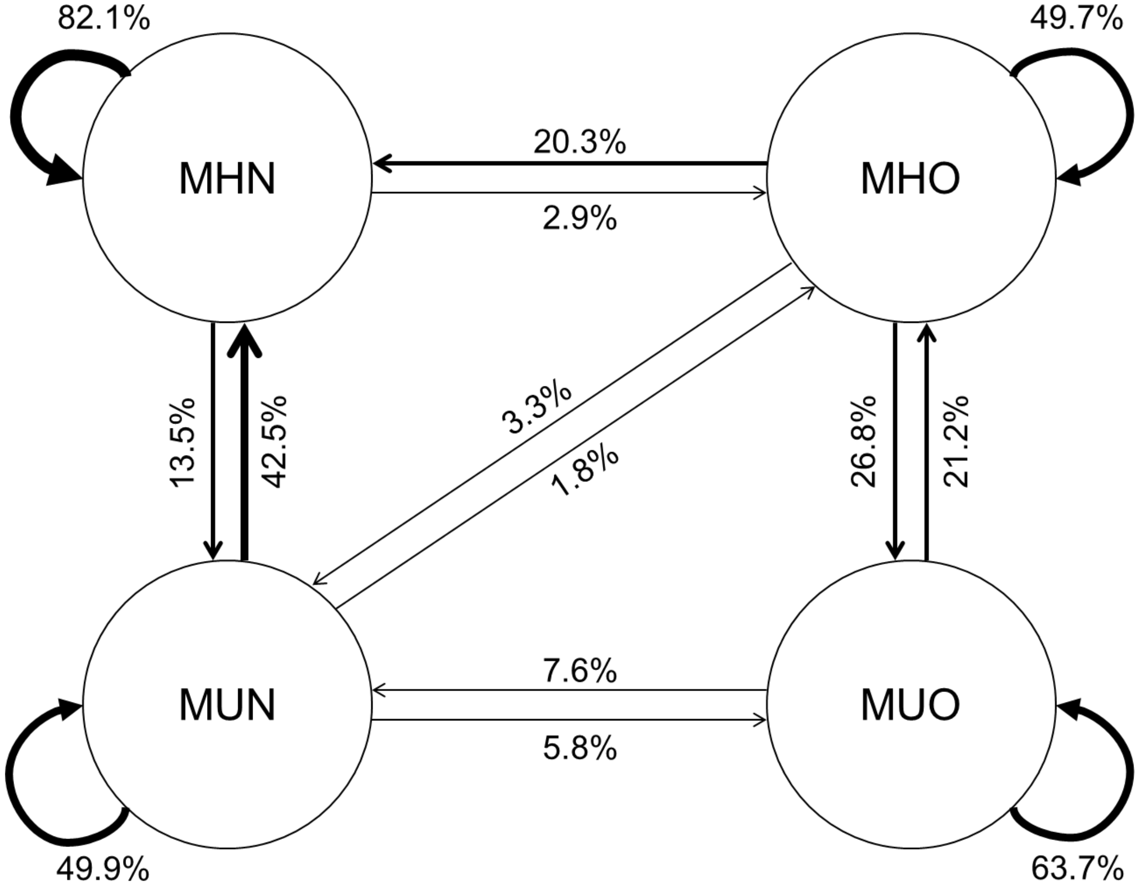

Supplement: Supplementary file 1 — (PDF 1198 kb) [file 125_2021_5484_MOESM1_ESM.pdf]
